# The CLEAR X-ray emission spectrometer available at the CLAESS beamline of ALBA synchrotron

**DOI:** 10.1107/S1600577522009821

**Published:** 2023-01-01

**Authors:** L. Simonelli, C. Marini, L. Ribo, R. Homs, J. Avila, D. Heinis, I. Preda, K. Klementiev

**Affiliations:** a CELLS-ALBA Synchrotron Light Source, 08290 Cerdanyola del Vallès, Barcelona, Spain; b MAX IV Laboratory, Fotongatan 2, 225 94 Lund, Sweden; ESRF – The European Synchrotron, France

**Keywords:** X-ray emission spectroscopy, innovative spectrometer, energy dispersive, wide and continuous energy range

## Abstract

The CLEAR X-ray emission spectrometer installed at the CLAESS beamline of the ALBA synchrotron is described. It is an energy-dispersive spectrometer based on Rowland circle geometry with 1 m-diameter circle. The energy dispersion is achieved by the combination of a diced analyzer crystal and a unidimensional detector. A single unconventional dynamically bent analyzer crystal (Si 111) permits a wide energy range to be covered, just by exploiting its different reflections (333, 444, 555, 777, 888): 6–22 keV, with a spectrometer efficiency that decreases above 11 keV because of the Si detector thickness (Mythen, 350 µm), while the relative scattering intensities for the Si 333, 444, 555, 777 and 888 reflections correspond to 36, 40, 21, 13 and 15, respectively. The provided energy resolution is typically below 1–2 eV and depends on the beam size, working Bragg angle and reflection exploited. In most cases the energy dispersion ranges from 10 to 20 eV and can be enlarged by working in the out-of-Rowland geometry up to 40 eV. The spectrometer works in full backscattering geometry with the beam passing through the two halves of the analyzer. The vacuum beam path and the particular geometry allow a typical average noise of only 0.5 counts per second per pixel. The spectrometer is mainly used for measuring emission lines and high-resolution absorption spectra, with a typical scanning time for highly concentrated systems of around half an hour, including several repeats. The intrinsic energy dispersion allows systematic collection of resonant X-ray emission maps by measuring high-resolution absorption spectra. Moreover, it allows spectra to be measured on a single-shot basis. Resonant inelastic X-ray scattering experiments to probe electronic excitations are feasible, although the spectrometer is not optimized for this purpose due to the limited energy resolution and scattering geometry provided. In that case, to minimize the quasi-elastic line, the spectrometer is able to rotate along the beam path. Advantages and disadvantages with respect to other existing spectrometers are highlighted.

## Abstract 2

The CLEAR emission spectrometer, a valid option with respect to other existing ones, is described.

## 1. Introduction

X-ray absorption spectroscopy (XAS) is nowadays widely exploited in the study of matter. Other cutting-edge synchrotron-based spectroscopic techniques such as X-ray emission spectroscopy (XES), X-ray Raman scattering (XRS) and resonant inelastic X-ray scattering (RIXS) are newer but are becoming more and more popular for the investigation of various systems.

Such hard X-ray spectroscopies are unique element-specific probes of the local electronic and atomic structure with a high level of complementarity. All of them are well suited for the characterization of all states of matter (from liquids to solids, crystalline, nanocrystalline or amorphous), which makes such techniques highly versatile. Moreover, the high penetration depth of hard X-rays and the typical XAS and XES measurement times easily allow *operando* or *in situ* experiments on batteries or catalysts (Fehse *et al.*, 2021 ▸; Mobilio, 2015 ▸; Zimmermann *et al.*, 2020 ▸; Timoshenko & Roldan Cuenya, 2020 ▸).

XAS provides both chemical and structural information at the local scale (4–5 Å) of the probed element (Bunker, 2010 ▸). XES is a photon-in/photon-out second-order process that follows the primary X-ray absorption, which generally focuses on dipole-allowed transitions by looking at the *K* fluorescence lines that are emitted after 1*s* core hole creation (Bergmann & Glatzel, 2009 ▸). While XAS gives access to the unoccupied electronic states of a selected element, XES probes the occupied ones. In both cases the spectral features depend on many entangled parameters (oxidation state, local structure, ligand character, spin state), and the coupling of the two techniques helps in identifying a unique and undoubted interpretation. Many examples can be provided. For example, the 3*d* states of transition metals govern their physical and chemical properties. Their electron distribution characterizes the absorption pre-peak in the X-ray absorption near-edge structure (XANES), whose shape and intensity depend on the oxidation state, spin state and local geometry. While the oxidation state and local geometry also affect the XANES and extended X-ray absorption fine structure (EXAFS) regions (Bunker, 2010 ▸), quantitative information on the local transition metal magnetic moment are accessible by XES, by measuring the *K*β emission line (Glatzel & Bergmann, 2005 ▸). On the other hand, EXAFS oscillations depend also on the nature of the ligand around the absorber (Carrera *et al.*, 2004 ▸). Several parameters simultaneously affect the EXAFS oscillations and it is well known that their correlations could lead to misleading interpretations. Moreover, ligands with very similar atomic number such as oxygen and nitro­gen are practically undistinguishable by EXAFS, and complementary information is needed to discriminate between them. XES provides such complementary information. Indeed, the *K*β′′ emission line in the valence-to-core spectral region shifts in energy depending on the ligand (Pollock *et al.*, 2014 ▸), independent of the atomic number.

Combined XAS and XES can thus provide the possibility of answering scientific questions when investigating complex systems. Moreover, the biggest advantage of such a multi-technique approach lies in the observation of dynamic processes, such as during *operando* or *in situ* experiments. This is where (quasi-)simultaneous data acquisition with different techniques ensures a perfect correlation between these measurements. As a result, information obtained using this approach goes beyond the sum that could be obtained by individual experimental methods, since a direct correlation can be applied.

Emission spectrometers technically allow other complementary inelastic X-ray scattering (IXS) techniques to be accessed, both in resonant and non-resonant mode (Moretti Sala *et al.*, 2018 ▸; Huotari *et al.*, 2017 ▸). RIXS is also an element-specific photon-in/photon-out second-order process that follows the primary X-ray absorption providing direct access to resonating electronic excitations (Lundberg & Wernet, 2019 ▸). Different from XAS and XES, it is a photon-thirsty technique where magnetic, *d*–*d* and charge transfer electronic excitations are enhanced by resonance. On the other hand, non-resonant inelastic X-ray scattering (NIXS) gives access to plasmons, Compton scattering and low-energy absorption edges with bulk sensitivity (XRS) (Wang & Zhu, 2020 ▸). Similarly to RIXS, NIXS and XRS are photon-thirsty techniques, which typically require high flux and spectrometer efficiency and long counting times.

At the CLAESS beamline of the ALBA synchrotron (Simonelli *et al.*, 2016 ▸), XAS and XES are simultaneously accessible in the 2.4–63.2 keV and 6–22 keV energy ranges, respectively. The CLEAR X-ray emission spectrometer available at CLAESS is optimized for XES measurements, but permits as well access to other IXS techniques. As at other beamlines (Moretti Sala *et al.*, 2018 ▸; Huotari *et al.*, 2017 ▸; Glatzel *et al.*, 2021 ▸; Ablett *et al.*, 2019 ▸; Hayama *et al.*, 2021 ▸; Kleymenov *et al.*, 2011 ▸; Llorens *et al.*, 2012 ▸), the CLEAR spectrometer is based on the so-called Rowland circle geometry which allows for simultaneous focusing and energy discrimination. In this geometry the source (sample), crystal analyzer and detector lie on the Rowland circle. The most common spectrometers used for collecting the emission signal with a high energy resolution in the hard X-ray regime are based on Bragg reflections from single or multiple perfect analyzer crystals in a Johann (Johann, 1931 ▸) or Johansson (Johansson, 1933 ▸) geometry. Different from the majority of the emission spectrometers available (Bergmann & Glatzel, 2009 ▸), the CLEAR spectrometer works for Bragg angles from 80° to 40° with a unique unconventional Johansson-like analyzer composed of several diced crystal stripes obtained by a unique Si ingot and mounted on a bender to focus in the direction perpendicular to the scattering plane. Moreover, the spectrometer works in total backscattering geometry relative to the sample, with the incoming beam passing through the two halves of the analyzer. All this is unusual. Indeed, for better energy resolution and focusing it is normally attempted to keep the Bragg angle close to 90° causing a relatively limited sample environment, due to the intrinsic proximity between sample and detector. Moreover, typically it is impossible to access extreme back- and forward-scattering geometries because of the intrinsic dimensions of the analyzer and its support. Additionally, it is normally necessary to change the crystal analyzer for each emission energy chosen, due to the limited range in energies per crystal reflection available. Finally, air paths and the windows of the sample setup chambers can be a strong source of background. All the above-mentioned characteristics affect the efficiency and versatility of this kind of instrument; the CLEAR emission spectrometer described here proposes a valid alternative.

## 2. Discussion

**Figure 1:**
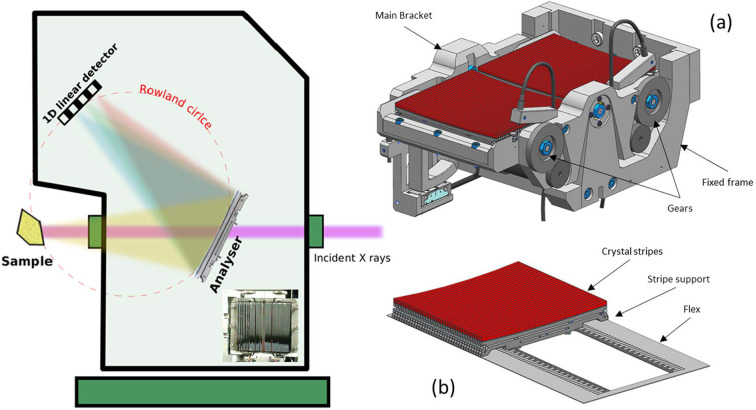
(Left) Schematic representation of the CLEAR spectrometer where the inset shows an image of the available Si(111) analyzer. (Right) Technical drawings of the Johansson-like CLEAR analyzer. (*a*) Full analyzer, where the main bender degrees of freedom are highlighted: the gears allowing the bending and the bracket permitting the scattered light on the detector to be aligned. (*b*) The flex on which the 72 crystal stripes glued on Al supports are mounted. The Al supports permit a fine angular alignment along the two directions perpendicular to the crystal surface.

The CLEAR X-ray emission spectrometer, designed for the CLAESS beamline of the ALBA synchrotron in Spain, eliminates all the aforementioned drawbacks; it is schematically shown in Fig. 1 ▸ (left). It is foreseen to work, like other existing emission spectrometers, in Rowland circle geometry, where the sample, analyzer and detector lie on a circle of 1 m diameter (*R* = 1). Five motors (two translations and one rotation for the detector, and one rotation and one translation for the analyzer) ensure that this condition is satisfied during the energy scans. A sixth motor allows the background coming from the sample environment to be minimized by rotating a set of slits, located at the emitted intensity entrance of the CLEAR vacuum chamber, during the energy scans. The slits at the entrance of the spectrometer block spurious signals which could come from eventual setup windows, while the vacuum beam path implies a very low noise contribution. When the sample setup is incompatible with the sample vacuum chamber a Kapton window is installed at the exit of the spectrometer to keep it under vacuum. In such cases no significant background increase has been detected. The six motors work in continuous mode during the energy scans to optimize the spectrometer efficiency. The spectrometer works in-vacuum (10^−2^ mbar) and its vacuum chamber can rotate around the beam axis to change the sample–analyzer–detector (SAD) scattering plane from vertical to horizontal. In addition, to permit minimization of the elastic signal, this feature provides flexibility, decreasing the space constraints for sample setups. Different from other spectrometers, CLEAR covers between 6 and 22 keV continuously by means of a single Si(111) analyzer crystal by exploiting its different reflections. A Johansson-like crystal has been chosen with respect to the more standard Johann-like crystal to provide at least three times better energy resolution and an increase by ∼150% in efficiency, when considering Bragg angles <80° (the smaller the angle, the bigger the difference in favor of the Johansson geometry), as was found by modeling with *xrt* software (Klementiev & Chernikov, 2014*a* ▸,*b* ▸). An image of the analyzer in use is shown in the inset of Fig. 1 ▸(left), with technical drawings on the right. The analyzer is composed of up to 72 crystal stripes glued on Al supports, permitting a fine angular alignment along the two directions perpendicular to the crystal surface, which are mounted on a flexor, and the latter on a bender. In the future, three other analyzers can be located in parallel to improve intensity, resolution or energy range. Only the selected analyzer sees the emitted light. A translation parallel to the crystal stripes allows the desired analyzer to be selected. The CLEAR analyzer collects the sample backscattered photons, exploiting a wide working Bragg angular range (40° < Θ_B_ < 80°). It is in principle possible to rotate the full spectrometer vacuum chamber by 180° around the vertical direction and to place it downstream of the sample to access full forward-scattering geometry. The energy resolution and photon intensity (focal properties) are ensured by the diced form of the analyzer (Huotari *et al.*, 2005 ▸) and the analyzer dynamical sagittal bending, respectively. A Mythen striped detector (Si chip of 6 mm × 80 mm × 0.35 mm, composed of stripes of 0.055 mm × 6 mm) allows a good efficiency up to around 11 keV, which drops in the high energy range because of the intrinsic Si wafer thickness (350 µm). The combination of diced analyzer and unidimensional detector allows for spectrum acquisition on a single-shot basis. Both the accessible energy windows and total energy resolution depend on the variable Bragg angle (40–80°), incoming energy bandwidth (different for Si 111 and Si 311 monochromators) and beam size in the SAD scattering plane direction (0.1 mm and 0.2 mm in the vertical and horizontal directions, respectively). In most cases, by moving the spectrometer far away from the sample, it is possible to enlarge the energy windows up to a maximum of 40–50 eV.

**Figure 2:**
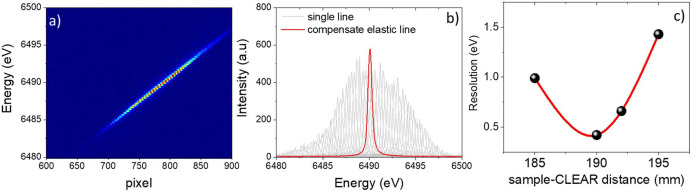
Elastic scan collected at the Mn *K*β emission energy. (*a*) Bidimensional map showing the evolution of the analyzer reflection on the detector as a function of the incoming energy. (*b*) Evolution of the analyzer reflection on the detector as a function of the incoming energy, where the pixel–energy conversion has been already applied. The red curve is the elastic line once compensated, *i.e.* the weighted sum of all the individual contributions shifted to overlap to the center of the scan. (*c*) Evolution of the energy resolution as a function of the sample–CLEAR spectrometer distance.

The main parameters affecting the CLEAR energy resolution are the choice of the incoming bandwidth (the selection of the Si 111 or Si 311 monochromator) and the beam size in the scattering direction, while the detector stripe (0.055 mm) or analyzer cube (1.4 mm) sizes marginally affect the energy resolution above 0.1 mm beam size or with Si 111 as the monochromator (with respect to the Si 311 monochromator, the Si 111 monochromator provides four time more intensity and a quarter of the energy resolution) (Verbeni *et al.*, 2009 ▸). The sample–CLEAR spectrometer distance also strongly affects the energy resolution and is optimized at the beginning of each experiment. In Fig. 2 ▸(*c*) the empirical evolution of the energy resolution as a function of the sample–CLEAR spectrometer distance is reported. The reported result shows how an enlarged probing depth above around 0.5–1 mm naturally broadens the accessible energy resolution. Moreover, attention is needed in comparing spectra of samples measured under the same geometrical conditions.

Once the sample–CLEAR spectrometer distance is optimized, the sample lies on the Rowland circle and the following formula allows the energy resolution to be estimated, considering all the above-mentioned parameters: (*A* ^2^ + *B* ^2^ + *C* ^2^ + *D* ^2^)^1/2^, where *A* corresponds to the incoming energy resolution (monochromator Darwin width), and *B* = (*s*/*R*)cot(Θ_B_), *C* = (*c*/*R*)cot(Θ_B_) and *D* = (*p*/2/*R*)cot(Θ_B_) correspond to the source (*s*), analyzer cube (*c*) and detector pixel (*p*) size contributions, respectively. The energy resolution and dispersion are measured for each experiment. As a scatterer for measuring the elastically scattered light, a tape foil is typically used. An example of such a scan obtained at the Mn *K*β emission line (6490 eV), by choosing the Si 311 monochromator and a beam size at the sample position of 0.1 mm, is reported in Fig. 2 ▸. Such a scan is performed by tuning the incident photon energy to around the energy selected by the CLEAR spectrometer in constant steps and recording the corresponding images on the Mythen detector. Use of a diced analyzer means different incoming energies are diffracted at different angles and to different pixels on the detector. It is possible to define a linear relationship between the energy and the pixels. Using such a relationship, it is possible to define the detector energy scale. Each incoming energy provides an elastically scattered line of which the full width at half-maximum (FWHM) corresponds to the global energy resolution. All the individual elastic lines are shifted and merged on the central one to provide higher statistics for estimating the energy resolution. The energy resolution (0.5 eV) corresponding to the scan reported in Figs. 2 ▸(*a*) and 2(*b*) matches the calculated one [see the Conceptual Design Report for CLEAR available online (Klementiev, 2009 ▸)]. The Si 111 monochromator provides around a factor of four higher intensity with quarter of the energy resolution compared with the Si 311 monochromator. This information needs to be considered at the beginning of each experiment to properly choose the monochromator according to the experimental requirements.

**Figure 3:**
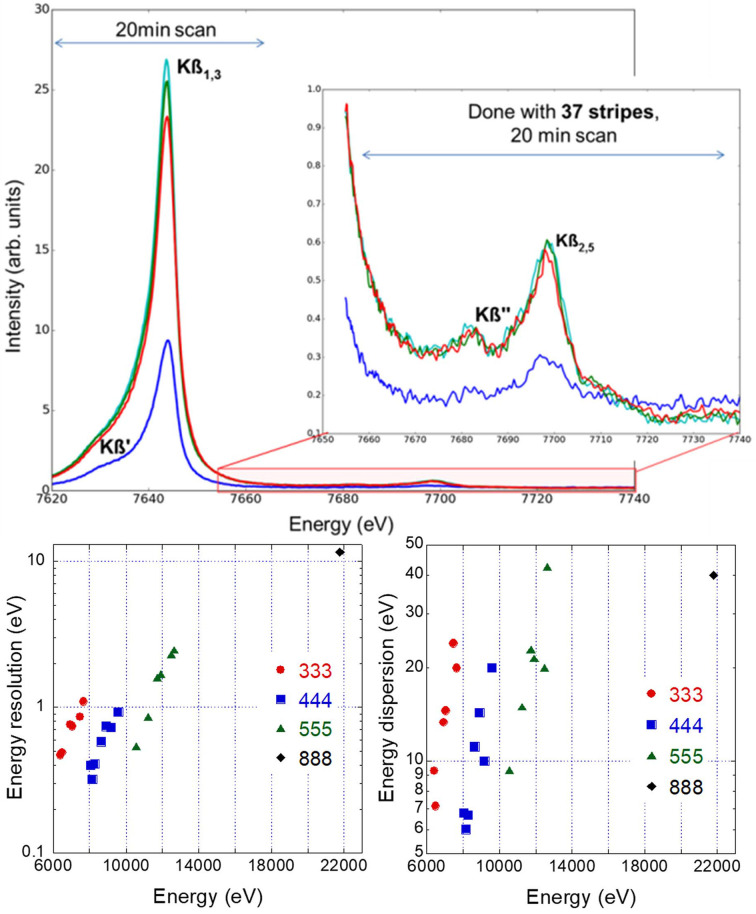
(Top) Representative Co *K*β emission spectra collected in 20 min for bulk samples (Li_*x*_CoO_2_ crystals, with different *x* content: 0.99, 0.66 and 0.46; the blue line corresponds to the signal collected for a pellet sample, while the others to signals obtained by measuring single crystals). (Bottom) Energy resolution and dispersion for several emission lines reachable with the Si 333, Si 444, Si 555 and Si 888 reflections empirically measured.

**Figure 4:**
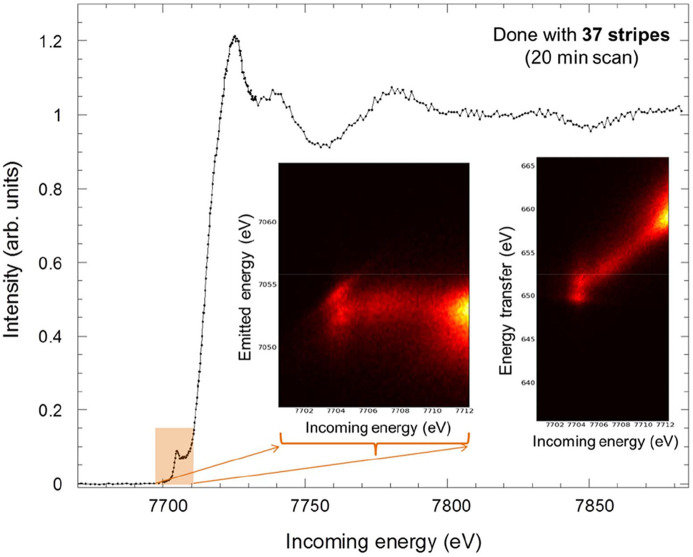
High-resolution Co *K*-edge X-ray absorption spectra, where the insets report bidimensional plots representing the spectral intensity as a function of the emitted (or transferred) and incoming photon energy in the pre-peak absorption region.

The energy resolution (FWHM of a quasi-elastic line collected for a tape foil) and dispersion (energy window seen by the spectrometer) for several emission lines measured for different elements (Ru, Ir, Ta, Kr, As, Se, Zn, Cu, Ni, Co, Fe and Mn) recorded with the Si 311 monochromator with a vertical beam size of 0.1 mm (vertical SAD scattering plane) in Rowland geometry are reported in Fig. 3 ▸, together with representative Co *K*β emission spectra collected for bulk materials. The reported emission spectra were acquired by scanning the outgoing energy at a fixed incoming energy. For each outgoing energy point the Mythen pixels correspond to a different energy scale. This needs to be compensated (aligning the energy scales and merging the corresponding intensity) to correctly visualize the spectra. This operation is carried out automatically during the experiments. Only 37 of 72 stripes are currently working, providing the statistics reported for around half an hour of acquisition time per *K*β emission line (including several repeats). The full backscattering geometry and the clean beam path under vacuum conditions allow any spurious background to be strongly suppressed down to only 0.5 counts per second with no emission signal. This, coupled with the intrinsic energy dispersion provided by the combination of a diced analyzer crystal and a unidimensional detector, permits the resonant X-ray emission (RXES) map to be acquired in a very short time, just by selecting the emission energy of interest using the CLEAR spectrometer and scanning the incoming energy across an absorption edge. As an example, Fig. 4 ▸ reports the high-energy-resolution absorption spectrum collected at the Co *K*-edge by selecting the Co *K*β emission line with a global energy resolution of 1.1 eV (FWHM of the quasi-elastic line), and the simultaneously acquired 2D RXES map covering the energy window seen by the spectrometer (energy dispersion around 20 eV).

More examples can be found in the literature. Since 2017 several experiments per year have exploited the CLEAR emission spectrometer, with some of the corresponding results, now published, in the majority of cases combining XES with XAS results.

**Figure 5:**
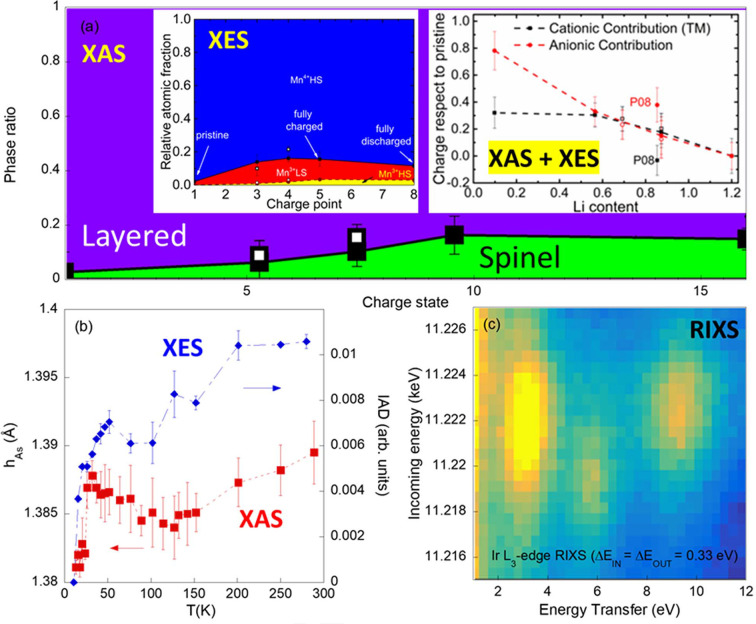
(*a*) The formation of a spinel phase at the expense of a layered phase in a Li- and Mn-rich cathode along the first charge quantified by Mn *K*-edge EXAFS. Mn *K*β emission spectroscopy of the averaged Mn local magnetic moment as a function of the charge state has been quantified and three Mn electronic phases have been detected. The local Mn magnetic moments have been extracted by the integral of the absolute difference [IAD method (Lafuerza *et al.*, 2020 ▸)] with respect to a reference. The union of the two results allowed access to the cationic contribution and, knowing the charge state indirectly, also to the anionic contribution, for the charge compensation mechanism (Ali *et al.*, 2022 ▸). (*b*) The chalcogen height and the local Fe magnetic moment relatively followed by the IAD with respect to the 10 K spectra in an Fe-based superconductor show a similar discontinuity across the superconducting temperature (around 30 K), providing clear evidence of the interplay between the electronic, lattice and magnetic degrees of freedom in these materials (Stramaglia *et al.*, 2021 ▸). (*c*) RIXS map collected at the Ir *L*_3_-edge over an Ir-based double perovskite compound with an energy step in both incoming and outgoing energy of 0.33 eV and a global energy resolution of around 1 eV (FWHM of the quasi-elastic line) (Bandyopadhyay *et al.*, 2019 ▸). Local and charge transfer electronic excitations are clearly visible around 3, 6 and 9.5 eV of energy transfer (brighter color).

XES can be considered still little exploited in the study of battery materials, despite its potential (Fehse *et al.*, 2021 ▸). In the first example, Mn *K*β XES has been successfully coupled to XAS at CLAESS to quantitatively identify the different Mn phases forming by charging and the full charge compensation mechanism by cycling in Li- and Mn-rich NMC cathodes (Simonelli *et al.*, 2019 ▸; Ali *et al.*, 2021 ▸, 2022 ▸) as schematically shown in Fig. 5 ▸(*a*).

Different from the battery field, IXS techniques are usually applied to the study of highly correlated systems to access details of their electronic properties. In the next examples, XAS and XES measurements collected at CLAESS are coupled to investigate Fe-based superconductors. In the first work, the results highlight how the suppression of superconducting transition temperature by Mn substitution in the fluorine-doped LaFeAsO is likely to be due to the competing Fe local magnetic moment (directly quantified by XES) and long-range magnetic correlations (Hacisalihoglu *et al.*, 2019 ▸). In the second, temperature-dependent Fe *K*-edge XAS and complementary Fe *K*β XES results obtained on a self-doped CaKFe_4_As_4_ superconductor reveal that both the anion height [red symbols in Fig. 5 ▸(*b*)] and the Fe local magnetic moment [blue symbols in Fig. 5 ▸(*b*), where the integral of the absolute difference (IAD) is expected to scale linearly with the Fe local magnetic moment] decrease sharply while the sample is cooled across the superconducting transition temperature. The concomitant decrease in the anion height while suppressing the local iron moment [Fig. 5 ▸(*b*)] provides clear evidence of the interplay between the electronic, lattice and magnetic degrees of freedom in these materials (Stramaglia *et al.*, 2021 ▸).

From the spintronics field, the electronic structure of perovskite oxide compounds such as doped LaFeO_3_ at the crossover from an insulating-to-metallic phase transition (Phuyal *et al.*, 2021 ▸) and half-metallic double perovskite Sr_2_FeMoO_6_ providing metallic conductivity and a gapped spin-up channel (Phuyal *et al.*, 2021 ▸) have been investigated by measuring the Fe *K*β emission line and Fe *K*-edge high-resolution absorption spectra by means of the CLEAR spectrometer.

Finally, the structural and physical properties of a series of Ir-based double perovskite compounds Pr_2–*x*_Sr_*x*_MgIrO_6_ (*x* = 0, 0.5, 1) were investigated using the CLEAR spectrometer (Bandyopadhyay *et al.*, 2019 ▸). An Ir *L*_3_-edge RIXS map, which has been collected on these systems at relatively low energy resolution (1 eV), is shown in Fig. 5 ▸(*c*). Thanks to the high *L*_3_-edge cross section, only around 3 h are needed for similar measurements on a bulk system. For weaker resonances the CLEAR efficiency is nowadays not competitive and long measurement times need to be considered. Completing the existing analyzer by exploiting the full available area is expected to provide around a factor of two higher efficiency. Moreover, changing the analyzer from diced to bent is expected to increase the collected intensity by a factor of ten by losing the energy dispersion.

## 3. Conclusions

The CLEAR X-ray emission spectrometer, available at the CLAESS beamline of the ALBA synchrotron, is an energy-dispersive spectrometer based on the Rowland circle geometry with 1 m-diameter circle. It works in-vacuum, in full backscattering geometry relative to the sample, in a wide Bragg angular range (40–80°) and with the incoming beam passing through the two halves of a dynamically bent diced analyzer. It exploits the Si 333, 444, 555, 777 and 888 reflections covering from 6 to 22 keV almost continuously, with a detector efficiency that decreases above 11 keV. The analyzer efficiency depends on the reflection exploited. Indeed, the relative scattering intensity for the Si 333, 444, 555, 777 and 888 reflections corresponds to 36, 40, 21, 13 and 15, respectively. The provided energy resolution is typically below 1–2 eV and depends on the beam size, working Bragg angle, and reflection exploited. The energy dispersion, provided by the diced analyzer and the Mythen uni­dimensional detector, ranges from 10 to 20 eV in most cases and can be enlarged by working in the out-of-Rowland geometry up to 40–50 eV. The spectrometer is optimized for measuring emission lines and high-resolution absorption spectra with a typical scanning time on highly concentrated systems of around half an hour, including several repeats. The intrinsic energy dispersion allows resonant X-ray emission maps to be systematically collected by measuring high-resolution absorption spectra. Moreover, it allows spectra to be measured on a single-shot basis. Resonant inelastic X-ray scattering experiments are feasible even though the spectrometer is not optimized for this purpose. The main advantage compared with other existing emission spectrometers lies in the wide continuous energy range which makes the instrument user friendly. The intrinsic complexity of the data post-processing is hidden from the users, as they get their data already reduced and corrected in their experimental data files. Moreover, the clean geometry and the working vacuum conditions are highly convenient to avoid spurious signals coming from the sample environment, a common problem for other spectrometers.

The main disadvantage is the limited efficiency due to the relatively small solid angle covered by the analyzer, which is not possible to easily expand, different from other existing spectrometers which typically exploit several analyzers in parallel. Finally, the space constraints induced by the vacuum condition limit the detector options.

Considering the above-mentioned advantages and dis­advantages, the CLEAR emission spectrometer is a valid option with respect to other existing spectrometers.
